# The Impact of the COVID-19 Pandemic on Mobility Trends and the Associated Rise in Population-Level Physical Inactivity: Insights From International Mobile Phone and National Survey Data

**DOI:** 10.3389/fspor.2022.773742

**Published:** 2022-03-14

**Authors:** Laurence J. Dobbie, Theresa J. Hydes, Uazman Alam, Abd Tahrani, Daniel J. Cuthbertson

**Affiliations:** ^1^Department of Cardiovascular and Metabolic Medicine, Institute of Life Course and Medical Sciences, University of Liverpool, Liverpool, United Kingdom; ^2^University Hospital Aintree, Liverpool University Hospitals National Health Service (NHS) Foundation Trust, Liverpool, United Kingdom; ^3^Institute of Metabolism and Systems, School of Clinical and Experimental Medicine, University of Birmingham, Birmingham, United Kingdom; ^4^Department of Diabetes and Endocrinology, Birmingham Heartlands Hospital, Birmingham, United Kingdom

**Keywords:** physical activity, COVID-19, mobility, exercise, green space, obesity, global health

## Abstract

**Introduction:**

The COVID-19 pandemic has reduced physical activity (PA) levels. This is important as physical inactivity is linked to poor COVID-19 outcomes. This study aimed to assess the impact of COVID-19 pandemic restrictions on greenspace and residence mobility, walking levels and in turn how these translated to trends in (UK) PA levels.

**Methods:**

Google Mobility Reports, the Oxford COVID-19 Government Response Tracker and Apple Mobility geospatial datasets were interrogated for international data. Residence mobility represents home mobility, greenspace mobility includes parks, walking direction requests is proportion of walking directions; stringency index measures lockdown intensity. The Sports England Active Lives Survey dataset was assessed for complementary changes in English PA levels.

**Results:**

Using mobility data of 10 countries we observed that during lockdown there were reductions in greenspace mobility and walking directions alongside increased residence mobility; more pronounced changes were seen in countries with higher stringency indices. From a UK perspective, complementary English PA survey data demonstrated the impact of these mobility changes on the proportion and demographic characteristics of PA levels. The most vulnerable in society, the elderly (ages 75+) and Black and Asian minority ethnicity (BAME) individuals were more likely to become physically inactive.

**Conclusions:**

The COVID-19 pandemic reduced greenspace mobility and walking direction requests globally. Complementary assessment of English PA levels demonstrated a greater proportion of the population became inactive. Demographics (75+ and BAME) prone to worse COVID-19 outcomes became disproportionately inactive. UK Urban planning should prioritize greenspace development. This could improve city walkability and PA levels.

## Introduction

COVID-19 was declared a global pandemic on 11th of March 2020 (Ma and Holt, 2020). As of 8th of September 2021, more than 223 million COVID-19 cases have been confirmed along with 4,605,021 COVID-19 related deaths (Caci et al., 2020; World Health Organization., 2020). Aside from the association between physical inactivity and non-communicable diseases such as type 2 diabetes, cardiovascular disease and cancer, physical inactivity is also an independent risk factor for COVID-19 severity. UK Biobank data (*n* = 3,87,109) has demonstrated physical inactivity is associated with increased risk of COVID-19 hospitalization and that daytime physical activity (PA) is associated with a reduced risk of severe COVID-19 illness (Hamer et al., 2020; Rowlands et al., 2020). Thus, PA appears to confer a degree of protection against COVID-19. Lockdown restrictions, however, have led to profound changes in mobility levels which may have resulted in reduced PA (Almandoz et al., 2020; Ruiz-Roso et al., 2020; Ong et al., 2021). For instance, working from home, stay at home orders and closure of leisure facilities may have reduced exercise levels; this might have contributed to, and exacerbated adverse outcomes of the pandemic. PA level reductions may also have been influenced by urban greenspace proximity (Mytton et al., 2012).

Previous research has highlighted that the COVID-19 pandemic has negatively affected PA level (Gallè et al., 2020; Meyer et al., 2020). In a large questionnaire of Brazilian individuals (*n* = 2,140) PA level reduced significantly during COVID restrictions when compared to pre-pandemic levels (Puccinelli et al., 2021). Similarly, two further Greek and Australian questionnaire based studies highlighted that PA level significantly reduced during the Spring COVID-19 lockdown (Bourdas and Zacharakis, 2020; Gallo et al., 2020). A Belgian study (*n* = 13,515) highlighted that the COVID-19 lockdown brought reductions in PA in those that were previously active, aged >55 years and of lower education status (Constandt et al., 2020). Similarly, an Italian study (*n* = 268) of patients with and without neuromuscular disease showed that total weekly PA reduced during the Spring COVID-19 lockdown (Di Stefano et al., 2021). A survey of 14 countries (*n* = 13,503) showed that compared to before the COVID-19 pandemic self-reported moderately vigorous physical activity (MVPA) reduced by 41% and vigorous physical activity (VPA) reduced by ~42%, with reductions greater for young and old rather than middle-aged individuals (Wilke et al., 2021). The effects of COVID-19 lockdowns on PA level is summarized by a systematic review highlighting across 64 studies that lockdowns reduced PA and increased sedentary behavior in a diverse range of populations (Stockwell et al., 2021).

It is important to understand how PA levels changed during this time, to help better inform future public health policies in general and in the event of a further pandemic. With this in mind, we conducted a study that aimed to assess the impact of the COVID-19 pandemic on global mobility using geospatial mobile phone data and to determine within England (using complementary physical activity survey data), how shifting PA patterns had influenced the proportions of the UK population who fulfilled the recommended daily PA level (to be considered *active* vs. *inactive*) from March 2020 until March 2021. We hypothesized that mobility during the pandemic has changed in proportion to lockdown stringency, a measure of lockdown intensity, and that as PA levels have concomitantly reduced, the proportion of the population who are inactive has increased. We were concerned about the demographic characteristics of those who have become most physically inactive (Tison Geoffrey, 2020; Ong et al., 2021).

## Materials and Methods

### Measure of Lockdown Restriction Intensity

#### Oxford COVID-19 Government Response Tracker

Oxford COVID-19 Government Response Tracker produced a stringency index measuring lockdown restriction intensity over the pandemic (https://www.bsg.ox.ac.uk/research/research-projects/coronavirus-government-response-tracker, accessed 31/03/2021). This index ranges from 0 to 100 based on the government's lockdown intensity (100 = most stringent restrictions). The nine metrics used to determine the index are: stay-at-home requirement, school closure, workplace closure, public event cancellation, public gathering restrictions, public transport closures, public information campaigns, internal movement restriction and international travel control. The index for each day is the mean score of the nine metrics, each metric is rated on a scale of 0 to 100. Analyses included countries (all members of the Organization for Economic Cooperation and Development) with high (India, UK, USA, Spain), medium (France, Germany) and low (Sweden, Australia, Japan, Finland) stringency indexes. These countries were chosen as they represent a broad spectrum of lockdown intensities (Hale et al., 2020).

### Measurements of Mobility: Global Data

Geospatial mobile phone data was obtained between February 2020 and March 2021.

#### Greenspace and Residential Mobility

Google Community Mobility Reports present mobility data which accounts for visits to, and length of stay at different geographical areas and compares it to baseline (https://www.google.com/covid19/mobility/, accessed 31/03/2021). This is anonymized data obtained from Google services like Google maps. Individuals were included in internationally collected data if they turned on location history settings, which by default is selected to be on in Google services. We examined two aspects of mobility: greenspace and residence mobility. Greenspace mobility represents mobility to areas including parks and national parks but not including the general outdoors i.e. rural areas. Residential mobility represents any proportion of mobility occurring within the home. Changes in these mobility domains were examined across 10 countries of varying stringency indexes. The baseline is a median value of the corresponding full day, i.e. 24 hour period, over a 5-week period (03/01/2020–06/02/2020). Therefore the baseline value is 7 individual values corresponding to that day's average over 5 separate 24 hour periods (Google LLC, 2020).

#### Walking Requests

Apple Mobility Trends provides Apple maps walking direction request data which represents the volume of direction requests compared to a baseline value on 13/01/2020 (https://covid19.apple.com/mobility, accessed 31/03/2021). This was calculated by counting the number of requests made to Apple maps for directions in the selected countries. For data to be available, a minimum number of requests had to be made to ensure user anonymity. The data provides no demographic information (COVID-19, 2020).

### Physical Activity: English Data

The Sports England Active Lives Surveys presents English data on active and inactive individuals (11/2019–11/2020) (https://www.sportengland.org/know-your-audience/data/active-lives/active-lives-data-tables, accessed 01/07/2021). The survey is sent to a randomly selected sample of households across England and includes a minimum of 500 people from each local authority. The survey is completed either electronically online or via a postal paper version. The survey takes ~15 min to complete. Each of the six surveys were over the previous 2 months (Range 15,163–39,041, *N* = ~29,372). The values presented represent % difference compared to 12 months previously. PA is defined as ≥10 min of moderately intense exercise. Active individuals were those exercising >150 min/week; inactive individuals were those exercising <30 min/week. Activity is based on moderate intensity equivalent minutes: each Vigorous intensity minute equates to two moderate minutes, each moderate intensity minute equates to one minute. Moderate intensity exercise was defined as exercise whereby breathing rate is elevated, vigorous exercise was defined as exercise whereby the person is sweating or out of breath. Supplementary Table 1 provides details of the questions used to determine physical activity level. Data was analyzed by Age, Sex, Ethnicity and National Statistics Socio-Economic Status (SES) which is an occupationally based classification ranging from group 1 (professionals) to group 8 (unemployed). The survey is a randomly selected sample of people in England with ~1,75,000 people taking part each year. There is a minimum of 500 people sampled in each local authority per year. The sampling is weighted to Office for National Statistics population measures in terms of demographics and location (Sport England, 2020).

### Statistics

Data was analyzed using R version 4.1.1 (R Foundation for Statistical Computing, Vienna, Austria). Data was tidied with the tidyr package. All graphs were created using the R package ggplot. Graphs were created for each measure separately, i.e: greenspace mobility, residence mobility, stringency index and walking direction requests. For measures of mobility, stringency index and walking direction requests this was calculated based on a 7-day rolling average. A descriptive analysis was then performed of mobility data by stringency index using the graphs. Survey data was analyzed based on sequential 2 monthly data, with active and inactive data split into separate graphs.

### Ethical Approval

All Google and Apple data used robust anonymisation techniques to ensure no individual could be identified. Therefore, no ethical approval was required.

## Results

### Measurements of Mobility: Global Data

#### Greenspace Mobility Trends and Relationship With Stringency Index

**Figure 1:**
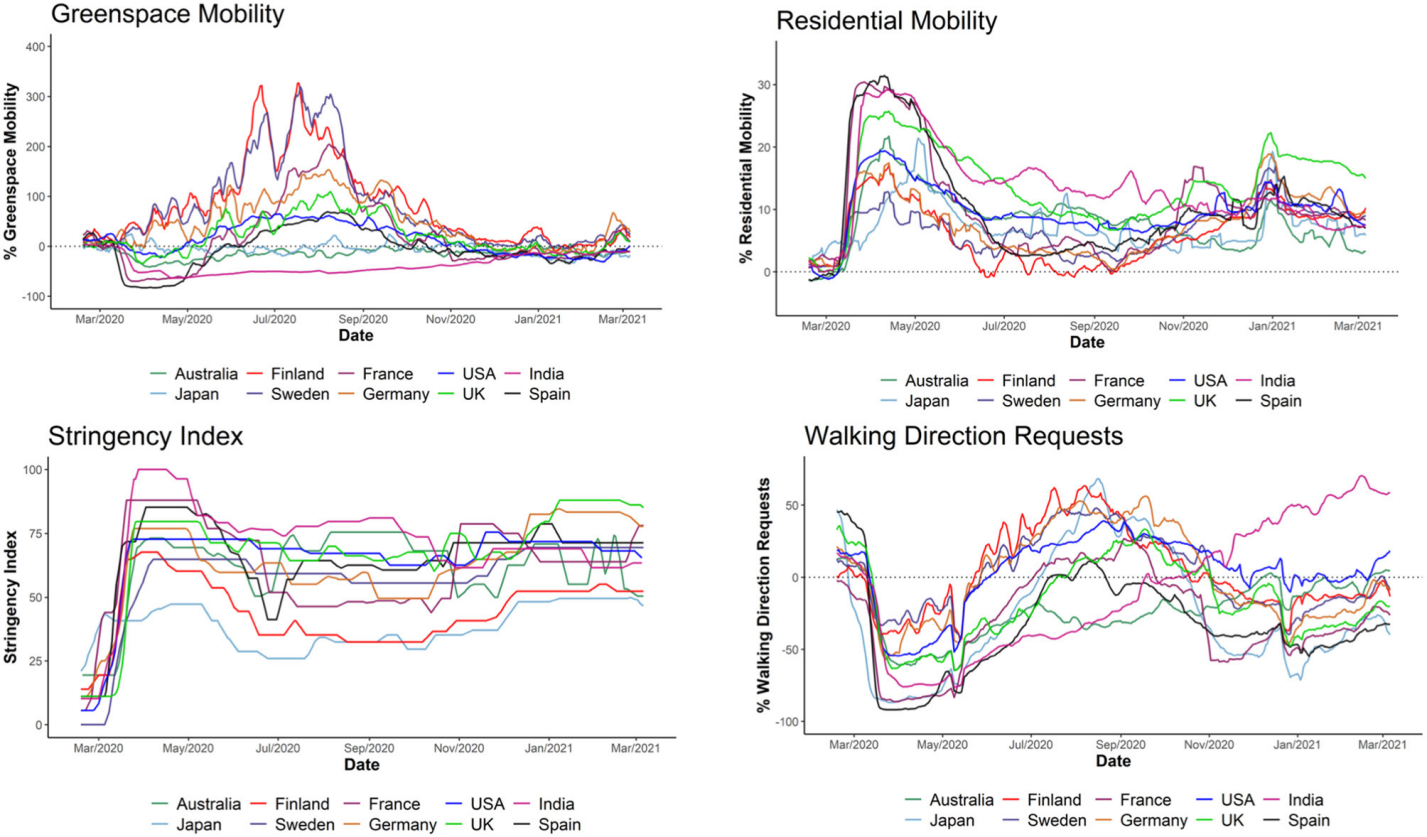
A comparison of mobility trends and stringency index for each country analyzed (Global Data). Google data (https://www.google.com/covid19/mobility/, accessed 10/03/2021) is % mobility. Apple data (https://covid19.apple.com/mobility, accessed 10/03/2021) presents walking direction requests. Oxford data (https://www.bsg.ox.ac.uk/research/research-projects/coronavirus-government-response-tracker, accessed 10/03/2021) describes the stringency index.

Figure 1 presents global mobility data including walking direction requests, greenspace mobility, residence mobility and Stringency Index. Finland, Sweden, Australia and Japan had a lower stringency index whereas UK, USA, India and Spain had a higher stringency index (Figure 1; Supplementary Tables S2–S5). Finland and Sweden trended to have the higher greenspace mobility across the pandemic. Most European countries (France, Germany, Sweden, Finland) had greater maximal greenspace mobility than the UK. Spain had a lesser maximal greenspace mobility than the UK, but also had a high stringency index. Outliers to greenspace trends were Japan and Australia. Japan had low SI but profound reductions in greenspace mobility. Australia had low SI, and large reductions in greenspace mobility, but to a lesser degree than Japan.

#### Residence Mobility Trends and Relationship With Stringency Index

All countries had increased residence mobility; a higher stringency index generally brought greater residence mobility whereas a lower stringency index generally brought lesser increases in residence mobility. Outliers to this trend were USA (high SI) and France (medium SI): despite the high SI USA had lesser increases in residence mobility than other high SI countries. In addition, France increased residence mobility to a similar degree as high SI countries.

#### Walking Direction Request Trends and Relationship With Stringency Index

All countries had reduced walking direction requests; a high stringency index brought generally greater and longer lasting reductions whereas a low stringency index generally brought smaller reductions in walking directions. Outliers to this trend were USA, France, Australia and Japan. Similar to residence mobility, USA had lesser reductions in walking directions and France had greater reductions in walking directions. Despite the low SI, Japan and Australia had greater reductions in walking directions than other low SI countries. Japan had greater overall reductions in walking directions than Australia.

#### UK Mobility Trends and Relationship With Stringency Index Obtained From Global Mobility Data

The March 2020 lockdown brought increased residence mobility alongside reduced greenspace mobility and walking direction requests (Figure 1; Supplementary Tables 2–5). These changes persisted until May when the stringency index reduced. Greenspace mobility and walking direction requests increased beyond baseline levels following lockdown, but trended down again during the second wave. Residence mobility consistently stayed above baseline.

### Physical Activity: English Data

#### English Physical Activity Surveys

**Figure 2:**
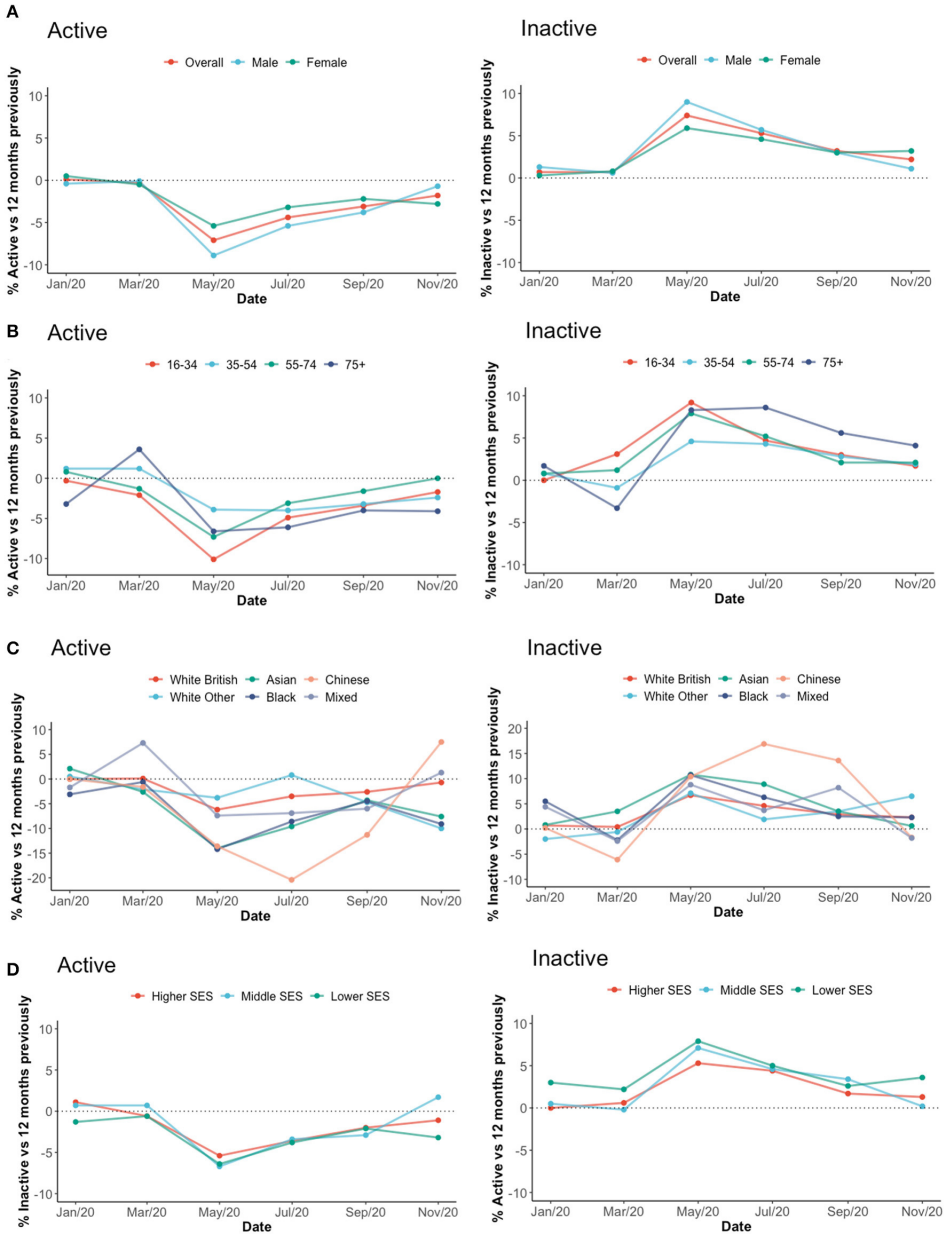
Trend in physical activity by sex, age and socioeconomic status (English data). This figure presents the Sports England Survey data (https://www.sportengland.org/know-your-audience/data/active-lives/active-lives-data-tables, accessed 01/07/2021) on % active (left-hand side) and inactive (right-hand side) individuals, SES, socioeconomic-status. **(A)** Sex, **(B)** age, **(C)** Ethnicity, **(D)** SES.

Between November 2019 and November 2020 the proportion of active individuals decreased and inactive individuals increased (Figure 2A Left Active, Right Inactive, Supplementary Tables 6, 7). Active individuals tended to be middle aged, female and Caucasian ethnicity; inactive individuals tended to be at the extremes of age and BAME individuals (Figures 2A–D). No obvious differences were noted between individuals with low and high SES.

#### English Physical Activity Levels Over 1st Lockdown, From English Survey Data

By comparing May 2020 to January 2020 the first UK lockdown brought a 7% reduction in active individuals with a 6.7% increase in inactive individuals (Figure 2A; Supplementary Tables 6, 7). Males, those aged 16–34 years and BAME individuals had the greatest decrease in PA and increase in inactivity (Figures 2A–C: Males −8.5% Active +7.7% Inactive, Age 16–34 −9.8% Active +9.2% Inactive, Asian −16.1% Active +10.0% Inactive, Black −11.1% Active +5.2% Inactive, Chinese −13.6% Active, +10.2% Inactive). Those aged 35–54 years, females and of Caucasian British ethnicity tended to maintain baseline PA and inactivity levels (Figures 2A–C: Age 35–54: −5.1% Active +3.8% Inactive, Females: −5.9% Active +5.6% Inactive, White British −6.2% Active +6.0% Inactive).

#### English Physical Activity Levels Over 2nd Lockdown, From English Survey Data

By comparing November 2020 to January 2020 the 2nd covid wave brought a 1.7% reduction in active individuals and a 1.5% increase in inactive individuals (Figure 2A; Supplementary Tables 6, 7). Females, those aged 75+ and from BAME backgrounds had greatest decreases in PA and increases in inactivity (Figures 2A–C: Female −3.3% Active +2.9% Inactive, Age 75+ −0.9% active +2.4% inactive, Asian −9.7% active−0.2% inactive, Black −6.0% active −3.2% inactive). Males and Caucasian British individuals maintained baseline PA levels (Figures 2A–C: Male −0.3% active −0.2% inactive, White British −0.7% active +1.6% inactive).

## Discussion

### Main Findings

This data shows that the COVID-19 pandemic has profoundly changed global mobility patterns. Within the UK individuals had a greater proportional reduction in greenspace mobility with reduced walking direction requests, compared to other European nations (France, Germany, Sweden, Finland). The suggestion from this data that the UK has become more physically inactive and been disproportionately affected by lockdown measures is borne out by complementary PA survey data from England. From the survey data, the proportion of individuals considered physical inactive (according to PA guideline recommendations) increased significantly, with the impact disproportionately affecting those of an older age and BAME ethnicity. This is important considering their association with adverse COVID-19 outcomes (Williamson et al., 2020) but also considering the known association of PA and even daily walking with long term outcomes (Saint-Maurice et al., 2022).

There was a correlation between higher stringency indices and shifting patterns of PA. This is exemplified by European nations (France, Spain, UK) with stringent restrictions during lockdown having the largest reductions in walking directions. Countries with the most stringent restrictions introduced penalties/fines for leaving the home without due reason. Future UK pandemic planning, however, should prioritize keeping the population active, which could involve greenspace development.

### Implications for Policy Makers

The study's data has implications for UK policy makers. Public health messaging around maintenance of PA should focus upon older age and BAME ethnicities and tackle weight change. Indeed, Public Health England reported that 32% of older people were inactive between March 2019 to May 2020, with average muscle strength and balance decreasing (Health Economics, 2021). This is important as physical deconditioning can predispose to mechanical falls and fractures in this demographic. In addition, recent Diabetes Prevention Program (DPP) data, which recruits a significant proportion of older individuals, highlighted an average weight difference of 2kg in those currently enrolling for the DPP vs. before the pandemic, indicating alarming trends in weight gain of the UK population (Valabhji et al., 2021).

These data also have implications for future UK urban planning which prioritizes greenspace development. Greenspaces provide exercise spaces which are free to all and naturally socially distanced and ventilated. Access to greenspaces has been shown to correlate positively with physical activity levels, and thus has the potential to improve outdoor exercise levels (Mytton et al., 2012; Gladwell et al., 2013; WHO., 2016; Wang et al., 2019). In addition, a systematic review demonstrated an inverse association between surrounding greenness and all-cause mortality, with the authors reporting the importance of greenspace implementation as a public health measure (Rojas-Rueda et al., 2019). Given the risk of future mutated COVID-19 strains and the independent association between physical inactivity and disease severity, the development of outdoor exercise spaces is vital. Wider access to greenspaces during future pandemic lockdowns would help our population to remain active whilst leisure facilities close. Overall, with the increasing incidence of obesity and related diseases within the UK, more effective UK urban planning may help improve PA levels and consequently reduce obesity prevalence and health inequalities (Mitchell et al., 2015; Google LLC, 2020; Hale et al., 2020; Sport England, 2020; COVID-19, 2020; NHS Digital., 2021).

### Comparison to Existing Data

Within the UK ~2.7 million people live over a 10-minute walk from a greenspace. By 2025 there will be ~6.5% increase in people living over a 10 minute walk from greenspaces (Green Space Index, 2020). This is concerning given those living closer to greenspaces tend to achieve recommended PA levels and have lower levels of diabetes and obesity (Mytton et al., 2012; Dalton et al., 2016; Zhang et al., 2018; de Keijzer et al., 2019). These facts signify the importance of UK urban planning in prioritizing greenspace development. This has potential to make the population more active (Mytton et al., 2012).

Previous research has highlighted that the COVID-19 pandemic has negatively affected PA level (Gallè et al., 2020; Meyer et al., 2020). Specifically survey research in Brazil, Greece, Australia, Belgium and Italy have all demonstrated PA level to have reduced (Bourdas and Zacharakis, 2020; Constandt et al., 2020; Gallo et al., 2020; Di Stefano et al., 2021; Puccinelli et al., 2021). In addition, a multinational survey of 14 countries (*n* = 13,503) reported that self-reported moderately vigorous physical activity (MVPA) reduced by 41% and vigorous physical activity (VPA) reduced by ~42% when compared to before the COVID-19 pandemic. These reductions were greater for the young and old rather than middle-aged individuals (Wilke et al., 2021). Our data has added benefit given the fact we show potential change in PA level over two analytical techniques. Specifically, the Sports England Active lives data shows in a large sample in England that PA levels reduced. In addition, global mobility data from 10 countries including the UK indicates that higher lockdown intensities led to lower levels of greenspace mobility, which could act as a proxy for PA in greenspace. This offers a specific strategy in that greenspace development must be prioritized to improve PA in future pandemic lockdowns.

Japan was a outlier to the data trends. Despite a low stringency index it had profound reductions in walking directions, little change in greenspace mobility and higher levels of residence mobility relative to other low stringency index countries (Finland, Sweden). This mirrors Japanese mobile phone Spatial Statistics data, reporting residence mobility increased in Japan during the Spring 2020 lockdown (Nagata et al., 2021). In addition, analyses using 2.00,000 anonymized Tokyo mobile phone users reported a ~50% reduction in mobility with lockdown, with this data strongly related to non-compulsory COVID restrictions. The authors even report a significant reduction in mobility before the spread of COVID-19 in Tokyo, supporting the populations cooperation with lockdown measures (Yabe et al., 2020). These data explain the trends observed, and could reflect cultural differences in Japan, whereby citizens are more compliant with lockdown measures than the other countries included in analysis. This may in part be related to Asia's previous experience with the severe acute respiratory syndrome (SARS) epidemic. Australia was another outlier, although to a lesser extent than Japan. This could be explained by it being in the Southern hemisphere, and thus the baseline data values which acted as comparison were in Australian Summertime, reducing this data's comparability when analyzed beside Northern hemisphere data.

### Strengths/Limitations

A major strength of this study is that we assessed ten countries using two large data sources and thus our findings are based on data from the mobility of millions of people from diverse countries. Secondly, we interrogated a national survey of English PA levels to support the impact of this geospatial data on our own population's PA levels. This facilitated assessment of how individuals' PA levels and the proportion of *active* vs. *inactive* individuals within the population have concomitantly changed reflecting the reduced mobility.

This dataset has limitations. The Google analyses are interpreted from a baseline value calculated within a 5-week period from January-February. This means the data may not be fully comparable to mobility data throughout the year. For instance, mobility will naturally vary with seasonal weather changes, i.e. individuals are generally indoors during winter. Working from home will have significantly impacted residence mobility, which will have contributed to the increased residence mobility reported. In addition, mobility data is a measure of location, meaning mobility to greenspace and residence may not directly correlate with activity and inactivity respectively. The apple dataset represents a subset of users who requested apple maps walking directions. A specific demographic could potentially use apple maps for walking meaning the data may not reliably estimate population PA levels. In addition, even when a walking direction was requested walking may not have taken place meaning this parameter may not directly relate to physical activity. Finally, apple and Google mobility data do not provide any demographic information and data about relative numbers of people it represents. This means we cannot state how this directly translates to the overall population. Survey data represents the views of individuals within England, meaning results may not be directly translatable to other UK nations. In addition the Survey data utilized self-reported PA. This is known to overestimate PA and underestimate physical inactivity when compared to objective measures of PA so may bias findings (Klesges et al., 1990; Jakicic et al., 1998; Tzetzis et al., 2001).

## Conclusions

The COVID-19 pandemic reduced global greenspace mobility and PA levels in England, with a greater proportion of the population not meeting the recommended PA guidelines and thus being considered physically inactive. Reduced exercise in greenspace could have contributed to the overall reduction of PA levels in England. Alarmingly, those with demographic characteristics (age 75+ and BAME ethnicities) prone to worse COVID-19 outcomes became disproportionately physically inactive. The work additionally highlights the role of greenspaces and the relevance of future UK urban planning in promoting physical activity and thus its importance in improving health-related outcomes across multiple chronic and infectious diseases including COVID-19.

## Data Availability Statement

The original contributions presented in the study are included in the article/Supplementary Material, further inquiries can be directed to the corresponding author.

## Author Contributions

LD, TH, UA, AT, and DC performed material preparation, data collection, and analysis. LD wrote the first draft of the manuscript and serves as the data guarantor. All authors contributed to the study conception and design, commented on subsequent versions of the manuscript, and read and approved the final manuscript.

## Conflict of Interest

The authors declare that the research was conducted in the absence of any commercial or financial relationships that could be construed as a potential conflict of interest.

## Publisher's Note

All claims expressed in this article are solely those of the authors and do not necessarily represent those of their affiliated organizations, or those of the publisher, the editors and the reviewers. Any product that may be evaluated in this article, or claim that may be made by its manufacturer, is not guaranteed or endorsed by the publisher.
