# Supplementary material for: The Impact of the COVID-19 Pandemic on Mobility Trends and the Associated Rise in Population-Level Physical Inactivity: Insights From International Mobile Phone and National Survey Data
Source: Front Sports Act Living. 2022 Mar 14;4:773742. doi: 10.3389/fspor.2022.773742 (PMC8967144; doi:10.3389/fspor.2022.773742)
Supplement: Supplementary file 1 [file Data_Sheet_1.docx]

**Supplementary Material: The impact of the COVID pandemic on mobility trends and the associated rise in population-level physical inactivity: insights from international mobile phone and national survey data**

Laurence J Dobbie^1,2^, Theresa Hydes^1,2^, Uazman Alam^3,4,5^, Abd Tahrani^6^, Daniel J Cuthbertson^1,2*^

1: Department of Cardiovascular and Metabolic Medicine, Institute of Life Course and Medical Sciences, University of Liverpool, Liverpool, United Kingdom

2: University Hospital Aintree, Liverpool University Hospitals NHS Foundation Trust, Liverpool, United Kingdom

3: Division of Diabetes, Endocrinology and Gastroenterology, Institute of Human Development, University of Manchester, Manchester, UK

4: Pain Research Institute and Department of Cardiovascular & Metabolic Medicine, Institute of Life Course and Medical Sciences, University of Liverpool and Aintree University Hospital NHS Foundation Trust, Liverpool, UK

5: Department of Diabetes and Endocrinology, Liverpool University Hospital NHS Trust, Liverpool, UK

6: Institute of Metabolism and Systems, School of Clinical and Experimental Medicine, University of Birmingham, Birmingham; Department of Diabetes and Endocrinology, Birmingham Heartlands Hospital, Birmingham, UK

***Correspondence**

Corresponding Author: Daniel J Cuthbertson

Department of Cardiovascular and Metabolic Medicine

Institute of Life Course and Medical Sciences

University of Liverpool

Liverpool

United Kingdom

E-mail: dan.cuthbertson@liverpool.ac.uk

ORCID of authors: Laurence J. Dobbie: 0000-0003-1908-848X

# Supplementary Data Tables

**Supplementary Table 1**: Questions used to determine Physical Activity Level in Sports England Active Lives Survey

|  | Question |
| --- | --- |
| 1 | (i)During which periods over the past 12 months have you done the following activities?  (walking, gardening, cycling, sport and fitness, and dance)  (ii) Timeframes in which that these activities have been undertaken: 7-12 months ago, 4-6 months ago, 1- 3 months ago |
| 2 | Have you done these activities in the past 4 weeks since XXXX? |
| 3 | During the past 4 weeks, on how many days did you do the activity? (Number of Days) |
| 4 | How much time did you usually spend doing that activity on each day that you did the activity? (Hours + Minutes) |
| 5 | Was the effort you put into the activity usually enough to: Raise your breathing rate? Make you sweat or out of breath? (yes/no) |

**Supplementary Table 2: Greenspace Mobility**

| **Country** | **02/20 (%)** | **03/20 (%)** | **04/20 (%)** | **05/20 (%)** | **06/20 (%)** | **07/20 (%)** | **08/20 (%)** | **09/20 (%)** | **10/20 (%)** | **11/20 (%)** | **12/20 (%)** | **01/21 (%)** | **02/21 (%)** | **03/21 (%)** |
| --- | --- | --- | --- | --- | --- | --- | --- | --- | --- | --- | --- | --- | --- | --- |
| **Japan** | 0.80 | 2.84 | -1.07 | -5.26 | -5.33 | -9.42 | 3.84 | 0.57 | -0.77 | 1.67 | -15.52 | -25.45 | -16.14 | -19.67 |
| **Finland** | 18.73 | 24.61 | 58.27 | 91.84 | 211.20 | 233.52 | 176.90 | 107.37 | 62.26 | 20.83 | 17.06 | 5.16 | 9.64 | 21.00 |
| **Sweden** | 7.53 | 18.77 | 64.20 | 93.84 | 191.53 | 239.06 | 213.13 | 91.33 | 41.13 | 13.17 | 6.29 | 5.10 | 17.39 | 26.56 |
| **Australia** | -0.13 | -13.71 | -35.77 | -24.58 | -14.97 | -12.32 | -13.65 | -5.90 | -8.13 | -11.77 | -8.87 | -4.55 | -16.25 | -12.78 |
| **France** | 20.07 | -31.74 | -63.90 | -14.13 | 37.57 | 133.23 | 162.00 | 61.97 | 13.10 | -24.90 | -14.84 | -22.45 | 1.54 | 14.22 |
| **Germany** | 10.33 | 10.13 | 38.93 | 53.16 | 88.57 | 116.90 | 121.16 | 100.77 | 44.84 | 12.83 | -6.81 | -8.00 | 20.00 | 32.56 |
| **USA** | 20.07 | -31.74 | -63.90 | -14.13 | 37.57 | 133.23 | 162.00 | 61.97 | 13.10 | -24.90 | -14.84 | -22.45 | 1.54 | 14.22 |
| **Spain** | 20.53 | -43.10 | -78.43 | -22.68 | 15.07 | 45.58 | 59.39 | 20.03 | -3.35 | -14.37 | -15.68 | -28.19 | -14.68 | -9.89 |
| **UK** | -1.87 | -1.26 | -18.67 | 26.03 | 38.60 | 70.68 | 84.52 | 61.90 | 18.32 | 2.40 | -3.00 | -14.35 | 1.11 | 13.67 |
| **India** | 2.73 | -17.87 | -56.53 | -58.65 | -51.60 | -50.90 | -51.84 | -45.30 | -41.55 | -34.20 | -17.23 | -13.42 | -13.14 | -10.67 |

Google data (<https://www.google.com/covid19/mobility/>, accessed 10/03/2021) on % mobility.

**Supplementary Table 3: Residence Mobility**

| **Country** | **02/20 (%)** | **03/20 (%)** | **04/20 (%)** | **05/20 (%)** | **06/20 (%)** | **07/20 (%)** | **08/20 (%)** | **09/20 (%)** | **10/20 (%)** | **11/20 (%)** | **12/20 (%)** | **01/21 (%)** | **02/21 (%)** | **03/21 (%)** |
| --- | --- | --- | --- | --- | --- | --- | --- | --- | --- | --- | --- | --- | --- | --- |
| **Japan** | 2.00 | 4.71 | 12.03 | 13.77 | 6.87 | 7.06 | 7.48 | 5.50 | 4.10 | 4.80 | 6.71 | 9.77 | 7.25 | 6.00 |
| **Finland** | 1.13 | 7.84 | 13.87 | 8.03 | 1.63 | 2.06 | -0.26 | 1.03 | 4.35 | 6.10 | 10.26 | 10.03 | 8.86 | 10.22 |
| **Sweden** | 0.93 | 5.77 | 9.70 | 7.10 | 3.80 | 6.48 | 2.16 | 2.70 | 4.29 | 8.30 | 11.81 | 11.55 | 9.18 | 9.22 |
| **Australia** | -1.20 | 5.23 | 18.23 | 13.00 | 9.20 | 9.23 | 9.97 | 8.33 | 7.23 | 4.87 | 5.42 | 7.13 | 4.89 | 3.56 |
| **France** | 2.00 | 15.68 | 28.27 | 17.26 | 7.47 | 3.87 | 4.48 | 2.00 | 5.55 | 14.20 | 10.90 | 9.74 | 9.21 | 8.56 |
| **Germany** | 1.27 | 8.06 | 14.03 | 8.84 | 5.67 | 3.71 | 2.84 | 1.77 | 4.74 | 8.03 | 12.55 | 13.13 | 11.46 | 9.56 |
| **USA** | -0.07 | 8.10 | 18.23 | 13.19 | 9.30 | 8.97 | 7.61 | 7.17 | 7.16 | 9.53 | 11.03 | 11.13 | 10.57 | 7.89 |
| **Spain** | -1.07 | 15.61 | 29.10 | 18.94 | 8.30 | 2.94 | 3.55 | 4.40 | 6.35 | 9.33 | 9.52 | 12.45 | 9.21 | 7.11 |
| **UK** | 1.27 | 9.26 | 24.60 | 20.52 | 16.63 | 11.97 | 8.94 | 7.67 | 10.03 | 13.53 | 14.52 | 18.42 | 17.04 | 15.44 |
| **India** | 0.40 | 10.39 | 28.53 | 21.87 | 14.73 | 15.77 | 13.06 | 13.47 | 11.61 | 10.17 | 10.81 | 10.35 | 7.04 | 7.44 |

Google data (<https://www.google.com/covid19/mobility/>, accessed 10/03/2021) on % mobility.

**Supplementary Table 4: Walking Direction Requests**

| **Country** | **02/20 (%)** | **03/20 (%)** | **04/20 (%)** | **05/20 (%)** | **06/20 (%)** | **07/20 (%)** | **08/20 (%)** | **09/20 (%)** | **10/20 (%)** | **11/20 (%)** | **12/20 (%)** | **01/21 (%)** | **02/21 (%)** | **03/21 (%)** |
| --- | --- | --- | --- | --- | --- | --- | --- | --- | --- | --- | --- | --- | --- | --- |
| **Japan** | 26.61 | -70.06 | -82.73 | -62.24 | -28.72 | 14.65 | 50.14 | 35.17 | -1.76 | -50.20 | -51.01 | -53.96 | -30.98 | -39.18 |
| **Finland** | 4.28 | -24.52 | -28.94 | -11.65 | 27.04 | 46.90 | 53.70 | 26.34 | 8.05 | -8.10 | -19.88 | -14.44 | -11.91 | -10.78 |
| **Sweden** | 11.08 | -17.23 | -23.30 | -16.30 | 15.76 | 38.40 | 43.50 | 29.17 | 18.00 | -14.56 | -23.24 | -20.58 | -11.72 | -7.15 |
| **Australia** | 13.76 | -24.61 | -57.42 | -49.91 | -29.67 | -24.39 | -33.06 | -26.79 | -21.87 | -15.84 | -6.15 | -6.86 | -3.90 | 2.39 |
| **France** | -2.83 | -54.12 | -83.32 | -58.02 | -17.61 | 8.99 | 13.18 | 15.86 | -11.58 | -56.50 | -43.08 | -41.94 | -29.93 | -25.92 |
| **Germany** | 18.66 | -26.82 | -34.63 | -18.51 | 14.84 | 40.06 | 44.21 | 45.75 | 26.99 | -13.93 | -28.07 | -28.06 | -15.65 | -7.57 |
| **USA** | 20.08 | -21.98 | -49.76 | -23.33 | 11.28 | 22.20 | 32.88 | 29.13 | 23.86 | 4.27 | 3.95 | -1.54 | 3.57 | 14.52 |
| **Spain** | 47.29 | -44.76 | -87.63 | -67.56 | -45.12 | -4.65 | 1.66 | -6.18 | -23.42 | -40.98 | -39.60 | -49.57 | -38.69 | -33.88 |
| **UK** | 30.86 | -20.57 | -57.50 | -45.79 | -31.57 | -5.90 | 15.65 | 24.56 | 7.49 | -21.14 | -19.21 | -36.61 | -26.64 | -20.15 |
| **India** | 15.60 | -27.06 | -74.80 | -65.48 | -46.43 | -40.33 | -29.77 | -9.72 | 3.36 | 21.14 | 40.22 | 51.28 | 62.57 | 56.60 |

Apple data (<https://covid19.apple.com/mobility>, accessed 10/03/2021) on walking direction requests.

**Table 5: Stringency Index**

| **Country** | **02/20 (%)** | **03/20 (%)** | **04/20 (%)** | **05/20 (%)** | **06/20 (%)** | **07/20 (%)** | **08/20 (%)** | **09/20 (%)** | **10/20 (%)** | **11/20 (%)** | **12/20 (%)** | **01/21 (%)** | **02/21 (%)** | **03/21 (%)** |
| --- | --- | --- | --- | --- | --- | --- | --- | --- | --- | --- | --- | --- | --- | --- |
| **Japan** | 26.23 | 41.16 | 45.74 | 41.90 | 27.78 | 27.99 | 32.71 | 33.06 | 33.04 | 36.67 | 46.89 | 49.36 | 49.64 | 46.91 |
| **Finland** | 15.00 | 45.10 | 63.95 | 55.88 | 39.51 | 36.62 | 33.58 | 32.41 | 35.90 | 41.27 | 51.47 | 52.31 | 53.50 | 52.31 |
| **Sweden** | 0.00 | 28.02 | 64.26 | 64.81 | 61.48 | 59.26 | 57.47 | 55.56 | 55.56 | 59.01 | 67.92 | 69.44 | 69.44 | 69.44 |
| **Australia** | 19.44 | 38.20 | 71.48 | 67.35 | 55.01 | 69.82 | 75.10 | 74.72 | 65.67 | 52.76 | 65.99 | 60.68 | 63.26 | 50.46 |
| **France** | 9.73 | 65.74 | 87.96 | 80.08 | 66.20 | 48.09 | 47.37 | 48.40 | 50.17 | 78.33 | 70.34 | 63.89 | 65.28 | 78.09 |
| **Germany** | 13.33 | 48.77 | 76.85 | 63.84 | 61.33 | 56.79 | 57.93 | 50.50 | 55.21 | 64.03 | 75.24 | 83.51 | 82.87 | 77.78 |
| **USA** | 5.56 | 45.77 | 72.69 | 72.69 | 70.71 | 68.26 | 67.13 | 64.20 | 64.05 | 69.63 | 71.88 | 71.76 | 68.32 | 65.74 |
| **Spain** | 11.11 | 51.73 | 85.19 | 78.58 | 54.17 | 59.48 | 62.80 | 61.02 | 64.67 | 71.30 | 73.45 | 72.73 | 71.30 | 71.30 |
| **UK** | 11.11 | 36.63 | 79.63 | 74.23 | 70.87 | 65.38 | 67.64 | 65.57 | 67.89 | 68.33 | 72.36 | 86.89 | 87.50 | 85.29 |
| **India** | 10.19 | 54.52 | 98.64 | 82.08 | 76.28 | 76.70 | 79.76 | 79.11 | 68.08 | 64.04 | 68.98 | 67.73 | 61.90 | 63.43 |

Oxford data (<https://www.bsg.ox.ac.uk/research/research-projects/coronavirus-government-response-tracker>, accessed 10/03/2021) on the stringency index.

**Supplementary Table 6: active Lives Active**

| **Date** | | **Nov/19 – Jan/20 (%)** | **Jan – Mar/20 (%)** | **Mar – May/20 (%)** | **May – Jul/20 (%)** | **Jul – Sep/20 (%)** | **Sep – Nov/20 (%)** |
| --- | --- | --- | --- | --- | --- | --- | --- |
| **Overall** | | 0.1 | -0.3 | -7.1 | -4.4 | -3.1 | -1.8 |
| **Sex** | **Male** | -0.4 | -0.1 | -8.9 | -5.4 | -3.8 | -0.7 |
|  | **Female** | 0.5 | -0.5 | -5.4 | -3.2 | -2.2 | -2.8 |
| **Age** | **16-34** | -0.3 | -2.1 | -10.1 | -4.9 | -3.4 | -1.7 |
|  | **35-54** | 1.2 | 1.2 | -3.9 | -4 | -3.2 | -2.4 |
|  | **55-74** | 0.8 | -1.3 | -7.3 | -3.1 | -1.6 | 0 |
|  | **75+** | -3.2 | 3.6 | -6.6 | -6.1 | -4 | -4.1 |
| **Ethnicity** | **White British** | 0 | 0.1 | -6.2 | -3.5 | -2.6 | -0.7 |
|  | **White Other** | 0.5 | -2.1 | -3.8 | 0.8 | -4.7 | -10 |
|  | **Asian (excl Chinese)** | 2.1 | -2.6 | -14 | -9.6 | -4.3 | -7.6 |
|  | **Black** | -3.1 | -0.6 | -14.2 | -8.6 | -4.5 | -9.1 |
|  | **Chinese** | 0 | -1.6 | -13.6 | -20.4 | -11.3 | 7.5 |
|  | **Mixed** | -1.7 | 7.3 | -7.4 | -6.9 | -6 | 1.3 |
| **SES** | **Higher** | 1.1 | -0.6 | -5.4 | -3.6 | -2 | -1.1 |
|  | **Middle** | 0.7 | 0.7 | -6.7 | -3.4 | -2.9 | 1.7 |
|  | **Lower** | -1.3 | -0.6 | -6.4 | -3.8 | -2.1 | -3.2 |

Sports England Survey data (<https://www.sportengland.org/know-your-audience/data/active-lives/active-lives-data-tables>, accessed 01/07/2021) on % active individuals, SES: socioeconomic status

**Supplementary Table 7: Active Lives Inactive**

| **Date** | | **Nov/19 – Jan/20 (%)** | **Jan – Mar/20 (%)** | **Mar – May/20 (%)** | **May – Jul/20 (%)** | **Jul – Sep/20 (%)** | **Sep – Nov/20 (%)** |
| --- | --- | --- | --- | --- | --- | --- | --- |
| **Overall** | | 0.7 | 0.7 | 7.4 | 5.3 | 3.2 | 2.2 |
| **Sex** | **Male** | 1.3 | 0.6 | 9 | 5.7 | 3 | 1.1 |
|  | **Female** | 0.3 | 0.8 | 5.9 | 4.6 | **3** | 3.2 |
| **Age** | **16-34** | 0 | 3.1 | 9.2 | 4.7 | 3 | 1.7 |
|  | **35-54** | 0.8 | -0.9 | 4.6 | 4.3 | 2.8 | 1.9 |
|  | **55-74** | 0.8 | 1.2 | 7.9 | 5.2 | 2.1 | 2.1 |
|  | **75+** | 1.7 | -3.3 | 8.3 | 8.6 | 5.6 | 4.1 |
| **Ethnicity** | **White British** | 0.7 | 0.4 | 6.7 | 4.6 | 2.9 | 2.3 |
|  | **White Other** | -2 | -0.6 | 7.1 | 1.9 | 3.5 | 6.5 |
|  | **Asian (excl Chinese)** | 0.8 | 3.5 | 10.8 | 8.9 | 3.5 | 0.6 |
|  | **Black** | 5.5 | -2.2 | 10.7 | 6.3 | 2.5 | 2.3 |
|  | **Chinese** | 0.2 | -6.1 | 10.4 | 16.9 | 13.6 | -1.7 |
|  | **Mixed** | 4.4 | -2.4 | 8.8 | 3.7 | 8.2 | -1.8 |
| **SES** | **Higher** | 0 | 0.6 | 5.3 | 4.4 | 1.7 | 1.3 |
|  | **Middle** | 0.5 | -0.2 | 7.1 | 4.6 | 3.4 | 0.2 |
|  | **Lower** | 3 | 2.2 | 7.9 | 5 | 2.6 | 3.6 |

Sports England Survey data (<https://www.sportengland.org/know-your-audience/data/active-lives/active-lives-data-tables>, accessed 01/07/2021) on % inactive individuals, SES: socioeconomic status
